# Nutrient Composition and Antinutritional Evaluation of Selected Wild Edible Plants Grown in Agroforestry of Simada District, Ethiopia

**DOI:** 10.1155/tswj/3545895

**Published:** 2025-05-01

**Authors:** Kindye Belaye Wassie

**Affiliations:** Department of Plant Science, College of Agriculture and Environmental Science, Bahir Dar University, Bahir Dar, Ethiopia

## Abstract

This investigation was carried out to evaluate the nutrient composition and antinutritional factors of five wild edible fruits *Embelia schimperi*, *Cordia africana*, *Ficus vasta*, *Mimusops kummel*, and *Syzygium guineense*. Proximate composition parameters (moisture, ash, crude fiber, crude fat, and crude protein) and antinutritional factors were evaluated using methods of the Association of Official Analytical Chemists and mineral analysis using the atomic absorption spectroscopy method. Microsoft Excel and Statistical Package for Social Sciences (SPSS Version 20) were used to analyze the data. Among the five wild edible fruit species, *Embelia schimperi* and *Cordia africana* had the highest fiber content (18 ± 0.03 g/100 g) and high protein content (8.7 ± 0.03 g/100 g), respectively. *Syzygium guineense* had the highest amount of moisture (16.3 ± 0.001 g/100 g), energy (320.58 ± 0.03 kcal/100 g), and carbohydrate content (76.72 g/100 g). *Embelia schimperi* was found to have the highest saponin content (2.1 mg/100 g). Oxalate content ranged from 0.46 mg/100 g in *Syzygium guineense* to 1.7 mg/100 g in *Cordia africana.* Mineral analysis showed that *Ficus vasta* had the highest Ca content (1015 mg/100 g), whereas the highest Zn content (38.6 mg/100 g) was investigated in *Embelia schimperi*. According to their recommended proximate and mineral contribution to daily nutrient requirements in humans, *Embelia schimperi*, *Cordia africana*, *Ficus vasta*, *Mimusops kummel*, and *Syzygium guineense* are sound in their dietary composition and in combating hunger especially in developing countries. More studies are vital to assess the nutritional composition and antinutritional quality of these wild edible plants and encourage farmers to cultivate in the agroforestry system.

## 1. Introduction

Wild edible plants provide vital supplements to the usual diets of cereal-based foods that are low in protein, minerals, and vitamins, especially for children living in rural areas [1]. Food and nutrition is one of the biggest problems facing the modern world which is suffering [2]. Numerous wild edible plants are excellent providers of nutrients that are sometimes lacking in typical diets, such as vitamins, minerals, protein, fat, and carbohydrate [3]. According to proximate analysis, the nutritional quality of wild edible plants is equivalent with or even better than that of domesticated forms [2]. Ash and water contribute to the mass of a creature, but proteins, lipids, and carbohydrates make up the entire energy content of an organism [4]. Antinutritional factor is produced in wild edible plant species during normal metabolism [5]. Wild edible plants greatly increase the food security of a family household and means of survival in times of famine [6]. The growing of wild edible plants in agroforestry holds the promise of achieving sustainable small-holder agriculture [7]. Agroforestry is a land use system that combines the disciplines of forestry and agriculture to generate a variety of goods in a specific amount of time and space, including food, fuelwood, fodder, lumber, and leaf litter [8]. Simada District is one of the districts found in Amhara Region, Ethiopia. The district is a food insecure area in Amhara Region, Ethiopia. The indigenous population frequently consumes wild edible plant species in periods of famine and normal times. There are plant species in this area that are extremely important for food security; however, there are not many scientific studies on the evaluation of the nutrient composition and antinutritional content of these edible wild plants. Therefore, it is necessary for ethnobotanists to evaluate the chemical composition of these wild edible plant species. The goal of this study was to evaluate the nutritional and antinutritional data on wild edible plant species that are used as food in Simada District, Ethiopia.

## 2. Materials and Methods

### 2.1. Plant Sample Collection and Preparation

**Figure 1:**
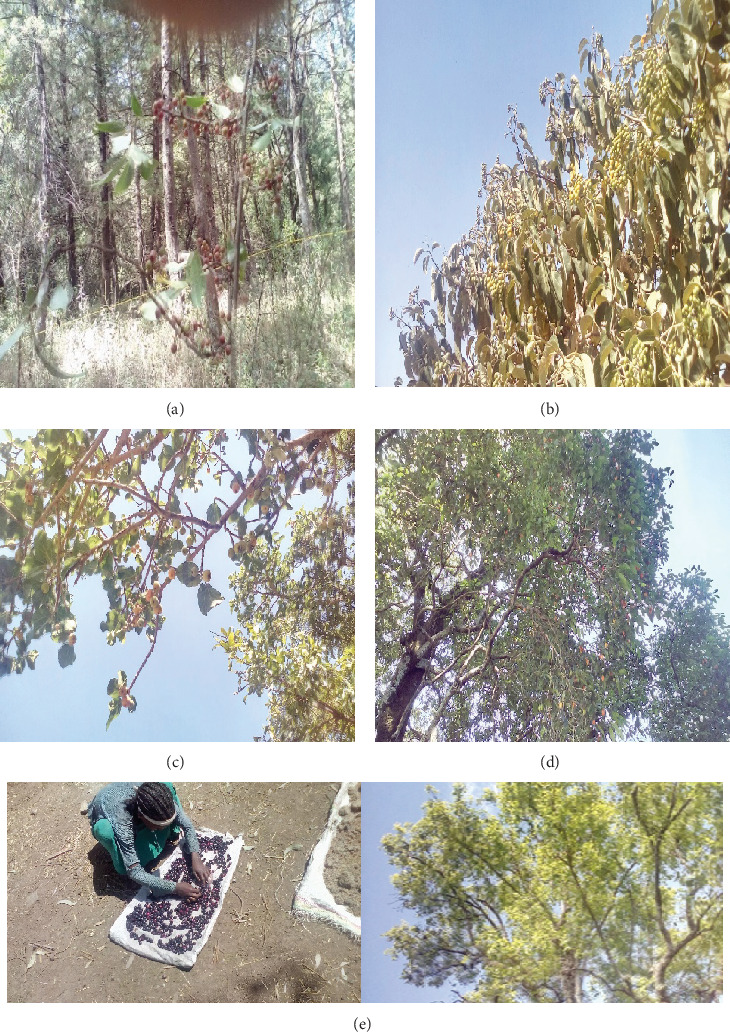
Semiwild fruit–bearing edible plant species for the present study: (a) *Embelia schimperi*, (b) *Cordia africana*, (c) *Ficus vasta*, (d) *Mimusops kummel*, and (e) *Syzygium guineense.*

This study was conducted in Simada District, South Gondar Zone, Amhara Regional State, located 11°29⁣′59.99⁣^″^ N latitude and 38°14⁣′60.00⁣^″^ E longitude. The district is about 774 km away from Addis Ababa and 209 km from Bahir Dar. The elevation spans from 1700 to 2600 m above sea level, with an average annual minimum and maximum temperature of 14–28°C and 200–900 mm of rainfall [9]. Fruits of plant samples *Embelia schimperi*, *Cordia africana*, *Ficus vasta*, *Mimusops kummel*, and *Syzygium guineense* were gathered from the agroforestry sites (Figure 1). The collected plant specimens were identified by taxonomists from Bahir Dar University using the flora book of Ethiopia and Eritrea [10]. A voucher specimen was placed in the herbarium of the Department of Plant Science Bahir Dar University. The fruit samples were cleaned with distilled water to remove any debris, gently dried at room temperature, and chopped into tiny pieces. The dried materials were pulverized to a fine powder using an automated motor blender, which was retained in airtight glass containers at 4°C until further analysis.

### 2.2. Proximate and Antinutritional Analysis

#### 2.2.1. Moisture Analysis

The moisture content of the fruit sample was determined according to the AOAC methods [11]. For each sample, 5.0 g of flour was placed in a crucible and dried in a 105°C oven until a consistent weight was achieved. The moisture analysis (%*M*) was calculated using the following formula:

(1) $$\begin{array}{c} \text{Moisture}\%=\frac{W3-W1}{W2-W1}\times 100, \end{array}$$

where *W*2 represents the weight of the sample and crucible, *W*3 represents the weight of the dry sample and the crucible, and *W*1 represents the weight of the empty crucible.

#### 2.2.2. Ash Determination

The AOAC standard procedure was used to calculate the amount of ash [11]. Two grams of each sample was added to porcelain crucibles, which were then weighed and burned at 550°C for 30 min. The samples were taken out and allowed to cool in a desiccator after being ashed. The percentage of ash is computed as follows:

(2) $$\begin{array}{c} \text{Ash}\%=\frac{W3-W1}{W2-W1}\times 100, \end{array}$$

where *W*1 is the weight of the empty crucible, *W*2 is the weight of the pattern and crucible, and *W*3 is the weight of the dry pattern and crucible.

#### 2.2.3. Determination of Fiber

The procedure described in [12] was used to calculate the crude fiber content. A 600-mL beaker containing 2 g of the sample was filled with 200-mL of 1.25% sulfuric acid and heated for 30 min. After 30 min of digestion in 20 mL of 28% NaOH, the mixtures were filtered through layer in a crucible using a vacuum pump. The leftovers were then repeatedly washed with hot distilled water. The remaining residue was vacuum-washed three times, using 30 mL of 1% sulfuric acid solution, distilled water, 1% sodium hydroxide solution, distilled water, and acetone in each wash. The residue was then dried using suction. After 2 h of drying at 130°C, cooling in desiccators, and measurement (*W*1), the samples were burned for 2 h at 550°C in a muffle furnace, after which they were cooled in desiccators and weighed once more (*W*2). The percentage of total crude fiber was stated as follows:

(3) $$\begin{array}{c} \text{Crude fiber}\%=\frac{W3-W1}{W2-W1}\times 100, \end{array}$$

where *W*1 is the weight of the empty crucible, *W*2 is the weight of the sample and crucible, and *W*3 is the load of the dry pattern and crucible.

#### 2.2.4. Determination of Fat Content

The Soxhlet extraction technique (Soxhlet), the official AOAC [13] method 45.01, was used to determine the amount of fat in the pattern. For the Soxhlet extraction tube of the apparatus, 2 g of each plant sample was weighed on filter paper (Whatman No. 2) and placed in a dry cellulose thimble inside the extraction tube, after which petroleum ether (40°C–60°C) was added. The samples were flushed for 6 h. The extraction was continuously observed for 6 h at the same time as being gently heated. After extraction, the solvent was recovered, and the flask containing the extracted fats was dried at 100°C for 10 min to evaporate any remaining ether. The flask was changed into then weighed after being allowed to cool to room temperature within the desiccator. The drying and weighing process was repeated until a constant weight was achieved. Ultimately, based on the weight of the material before and after extraction, the percentage fat content of the material was determined. The fat content was calculated as a percentage of the sample weight using the following formula:

(4) $$\begin{array}{c} \text{Crude fat}\%=\frac{W3-W1}{W2-W1}\times 100, \end{array}$$

where *W*1 is the sample weight in grams, *W*2 is the extraction thimble weight in grams, and *W*3 is the extraction thimble weight in grams with the dried crude fat.

#### 2.2.5. Determination of Crude Protein

The micro-Kjeldahl method was used to evaluate crude protein content [14]. Five grams of fruit flour was added to a digestion tube along with three digestion pills and 15 mL of 98% pure H_2_SO_4_. After 3–4 h, digestion was stopped and a distinct green color was obtained. Next, a 100-mL conical flask with its outlet tubes inserted into the conical flask was placed beneath the distillation equipment. It contained 20 mL of 40% boric acid and three drops of Tashiro's indicator. After adding three drops of phenolphthalein and 20 mL of 40% (*w*/*v*) NaOH solution, the digest was removed with distilled water. The distillation process was then carried out again until approximately 50 mL of the distillate was trapped in the boric acid plus indicator solution, which turned light gray instead of red, signifying that all the ammonia released had been trapped. Using 0.1 mM HCl, a receiving flask was titrated until it turned brown. The percentage of nitrogen was determined as follows using titration:

(5) $$\begin{array}{c} \text{Protein}\%=\frac{6.25\left(V-Vb\right)\times N\times 14\times 100}{500}, \end{array}$$

Crude protein = %nitrogen × 6.25,

where *V*1 is the amount of acid (milliliter) needed to titrate the sample, *V*2 is the volume (milliliter) of HCl needed to titrate the blank, *M* stands for acid molarity, *N* is for HCl normalcy, 6.25 is for protein nitrogen conversion factor, and 1.4 is the nitrogen atomic mass.

#### 2.2.6. Total Carbohydrate Determination

The carbohydrate amount was calculated following [15]. Total carbohydrate was determined by subtracting the percentages of %moisture, %ash, %protein, and %fat content from 100. Total carbohydrate = 100 − (%) moisture + %ash + %crude protein + %crude fat + %crude fiber.

#### 2.2.7. Determination of Caloric Value

The caloric value of the tested plant sample was calculated following [16]. The energy value is equal to (4 × *P*) + (9 × *F*) + (4 × *C*), where *P* represents the protein content (percentage), *F* the fat content (percentage), and *C* for available carbohydrate (percentage).

#### 2.2.8. Calculating the Mineral Content

A sample of 2 g of dry fruit was scorched on a hot plate and then burnt for 3 h at 550°C in a muffle furnace until smoking stopped. The resulting white ash was weighed, dissolved in 3 mL of strong nitric acid, and then diluted with up to 25 mL of deionized water. Standard stock solutions of Ca, Zn, and Fe were created using grade standards from atomic absorption spectroscopy [17]. Using an acetylene light at wavelengths of 422.7, 213.9, and 248.3 nm, respectively, the minerals Ca, Zn, and Fe were identified through the application of an atomic absorption spectroscopy technique. Different sets of electrode lamps were used for each mineral. To make sure the apparatus was running correctly, it was run for standard solutions of each mineral both before and during the determination. To assess any potential contamination, blank solutions were prepared using the same chemicals and methodology as the samples and standards.

### 2.3. Determination of Antinutritional Factors

Antinutritional factors are substances that work to lower nutrient levels, use, bioavailability, or consumption of food. They have essentially a significant impact on restricting the broader use of numerous plants. They consist of phytate, alkaloids, tannins, cyanogenic glycosides, oxalate, and saponin [18]. The saponin content was ascertained using the double extraction gravimetric method, as outlined by [19]. The phytate content was ascertained using the technique in [20]. The tannin concentration was established applying techniques outlined by [21]. Oxalate content was determined by a method in [12]. After grinding the material (1 g), 75 mL of 3 mol/L H_2_SO_4_ was poured to a conical flask and agitated with a magnetic stirrer for an hour. After filtering, 25 mL of the filtrate was collected and heated to 80°C–90°C. The hot aliquot was continually titrated with 0.05 mol/L of KMnO_4_ after reaching the endpoint, as indicated by a light pink color lasting 15 s. To calculate the oxalate content, use the assumption that 1 mL of 0.05 mol/L KMnO_4_ equals 2.2 mg of oxalate.

### 2.4. Data Analysis

The results of the nutrient content and antinutritional investigations of wild edible fruit–bearing plants were inspected by one-way analysis of variance (ANOVA) methods; the outcome was presented as mean ± SE of three replications and was determined statistically significant using SPSS Version 20.

## 3. Results and Discussion

### 3.1. Taxonomic Information of the Specimens

The collected species of wild edible plants were found in different families (Table 1). Taxonomic information of *F. vasta* at the family level was obtained in a previous study [12] which states the nutritional and antinutritional content of *Ficus sur* in the Mekdela District, South of Wollo, Ethiopia, at the same family level with different species. The classification of plants based on their chemical components may be useful in the discovery of new culinary and medicinal plants as well as in the resolution of some taxonomy issues [22]. Plants within a taxon commonly exhibit similar metabolite content and bioactive qualities, and secondary metabolites are generally similar among members of a clade [23].

### 3.2. Proximate Composition

Table 2 shows the moisture content, ash, fat, protein, fiber, carbohydrate, and energy value of five wild edible plants. Among the studied wild edible plants, *S. guineense* had the highest moisture contents (16.3 ± 0.001 g/100 g) and carbohydrate content (76.72 ± 0.01 g/100 g) whereas it was found to have low total ash and crude protein. This result is higher than the previous scenario in Ethiopia [22]. The difference in the proximate content of the wild edible plants might be primarily based on the variations in the drying degree, the adulthood degrees of the fruit, and the growing areas and time of harvest and postharvest handling of the wild fruits. For instance, prolonged storage may lead to nutrient degradation, especially in moisture-sensitive components. The ash content ranged from 0.78 ± 0.00 g/100 g in *S. guineense* to 7.8 ± 0.002 g/100 g in *F. vasta*; this wide variation underscores the diversity of mineral content among wild fruits and highlights the potential for addressing micronutrient deficiencies through their consumption. The ash content of the current finding disagrees with the previous study of *Ziziphus nummularia* and *Ziziphus mauritiana* that are conceded out in India [24]. The amount of fiber in the current finding ranged from 3.4 ± 0.02 in *S. guineense* to 19.5 ± 0.00 g/100 g in *M. kummel*. This agrees to nearly 5%–22% of the recommended daily allowance (RDA) of fiber for humans, which is 19–38 g [25]. Compared to other studies reported by [12] in Ethiopia, the fat content ranged from 0.5 ± 0.01 g/100 g in *S. guineense* to 3.2 ± 0.01 g/100 g in *F. vasta*, which was higher value. The variation of fat content in the wild edible plants could be the physiological nature of the plant ecology and mineral availability of the plants. The highest and lowest protein content of the fruits was discovered, with *C. africana* (8.7 ± 0.03 g/100 g) and *S. guineense* (2.3 ± 0.01 g/100 g). The amount of protein content in the fruits of *C. africana* is comparable with previous finding [2], who reported the protein content of *P. laticoronum* (8.1 ± 0.4 g/100 g). The endorsed dietary allowance (RDA) of protein for a wholesome adult with minimal bodily activity is presently 0.8 g protein per kilogram body weight consistent with day [8]. The protein content difference between plant samples could be because of various environmental factors like soil type, nutrient availability, and moisture levels which can have a substantial impact on protein composition. Plants grown in nutrient-rich soils might have increased protein content. The average carbohydrate content ranged from 53.5 g/100 g g/100 g in *F. vasta* to 76.72 ± 0.01 g/100 g in *S. guineense*, which corresponds to approximately the RDA of carbohydrates (130–210 g/day) for all age groups [26]. This indicates that fruits can supply adequate amounts of the body's primary energy source. In the current findings, the energy value of tested wild edible plants ranged from 267.7 ± 1.3 kcal/100 g in *M. kummel* to 320.58 ± 0.03 kcal/100 g in *S. guineense*. Comparable results were discovered in the previous finding [12] in Ethiopia. The increased carbohydrate content in *S. guineense* could be due to differences in altitude where the gathered wild fruits grow, and the food may include a large amount of carbohydrate when the plant grows in nutrient-rich soils.

### 3.3. Mineral Composition

**Figure 2:**
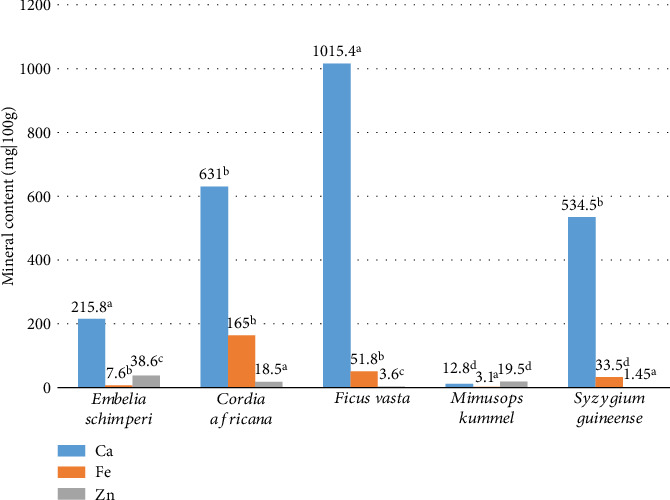
Mineral composition of wild edible plants in dry weight basis, the same superscript letters in the graph are significant differences at *p* < 0.05.

In this finding, the content of macromineral (Ca) and trace minerals (Fe and Zn) was examined in terms of dry matter (Figure 2). The highest value of calcium content was observed in *F. vasta* fruits (1015.4 mg/100 g) among the studied wild edible plants, while the lowest value of calcium was examined in the fruits of *M*. *kummel* (12.8 mg/100 g). The highest calcium content in the fruits of *F. vasta* could be a result of combination effects of plant's ability to uptake nutrients from its environment and its inherent genetic makeup. In this finding, the estimated calcium content was higher than the previous reported value (754.9 mg/100 g) by [12]. This could be difference in growing area, soil type, and climatic condition of wild edible fruits. The recommended nutritional requirement of calcium for women between 19 and 65 is 1000 mg [15]. The highest and lowest iron content was investigated in the fruits of *C. africana* (165 mg/100 g) and *M*. *kummel* (3.1 ± 0.01 mg/100 g). Higher iron content in the fruits of *C. africana* than the rest of the plant sample could be different plant species in their ability to absorb and accumulate iron, and some species may have more effective strategies for iron intake that increase nutrient absorption. In the current finding, a higher amount of iron was discovered than in earlier investigation [15], which reported a range of 7 mg/100 g–119 mg/100 g of calcium in pepper and spinach, 0.13 mg/100 g–1.89 mg/100 g of iron in lettuce and spinach, and 0.1 mg/100 g–0.90 mg/100 g of cucumber and spinach in Turkey. Iron is essential to sustain well cells, membrane, fur, and pins. Iron breakdown in the body is a multifarious progression that is controlled by hormones [16]. It also plays a vital part in numerous natural processes of human physiology, such as blood fusion, mitochondrial breathing, and catalytic responses [17]. Furthermore, the highest amount of zinc was recorded in the fruits of *E. schimperi* (33.6 mg/100 g). Zinc is an indispensable micronutrient known to play an energetic role in army protection beside disease [18]. Zinc association with protein synthesis is fundamental to various bodily functions. Proteins are essential for the structure, function, and regulation of the body's tissues and organs. Zinc functions as a cofactor for numerous enzymes involved in protein synthesis, facilitating the process of translating genetic information into functional proteins. Without adequate zinc levels, the body may experience disruptions in protein synthesis, potentially leading to impaired growth and development [27]. The daily intake of zinc was limited to 3–5 mg daily [19]. Variations in mineral content between plant samples could be influenced by species, soil type, environmental factors, and growing conditions. The calcium content of various plants is mostly determined by their ecological niches and the availability of this crucial nutrient in their growth habitats.

### 3.4. Antinutritional Determination

**Figure 3:**
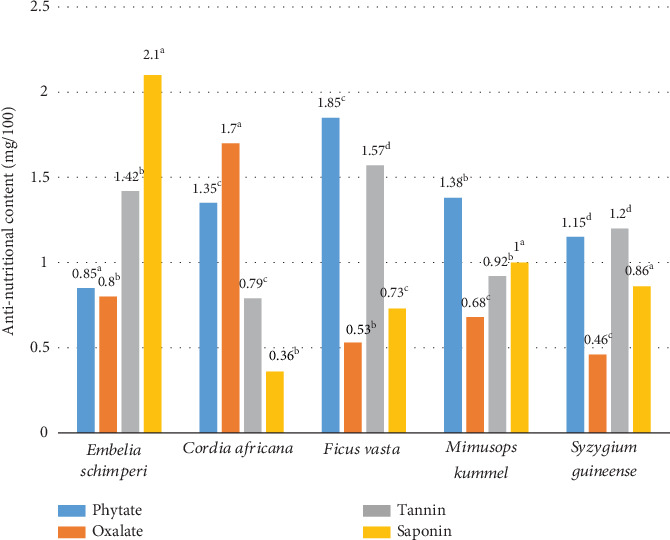
Antinutritional content of semiwild edible plants, the same superscript letters in the graph are significant differences at *p* < 0.05.

*F. vasta* had the highest phytate content (1.85 mg/100 g), while *E. schimperi* contains the lowest content (0.85 mg/100 g) (Figure 3). The amount of phytate in the current finding had a lower value than the previous study [28], who reported that the phytate content ranged from 6.9 ± 0.00 mg/100 g for *Gardenia ternifolia* to 51.4 ± 0.041 mg/100 g for *Clausena anisata*. This variation could be difference in the plant's maturity during harvest can also influence phytate levels. In general, the phytate content of fruit might alter as they mature and prepare for storage. Phytate is a regular portion known to be an antinutritional constituent in Fabaceae and observed as a key storage complex for phosphorus [21]. Oxalate content ranged from 0.46 mg/100 g in *S. guineense* to 1.7 mg/100 g in *C. africana.* Oxalate is a naturally occurring compound found in many foods, such as spinach, rhubarb, and almonds. High levels of oxalate in the diet have been associated with the formation of kidney stones, as oxalate can bind with calcium to form insoluble crystals. In addition to its role in kidney stone formation, oxalate has also been shown to inhibit renal calcium absorption [29]. The intake of extra oxalic acid may lead to pebble establishment in the bladder after the acid is defecated in the urine [30]. Nevertheless, the standards established in this analysis were meaningfully inferior to the standards that are measured harmful. This suggests that consumption of ripened ovary could not be the reason of somewhat difficulties through the uptake of nutrients over human parts [22]. In the realm of dietary habits and their effects on mineral absorption, the relationship between fruit consumption and mineral intake has long been a topic of interest. The notion that eating fruits may not interfere with the body's ability to absorb minerals is a subject that warrants further exploration and discussion [22]. The tannin content in the present study was between 0.79 mg/100 g in *C. africana* and *F. vasta*, respectively. The greater breakdown and the greater competence in exploitation of nutrients encouraged by adding of tannins to diets take remained endorsed to their capacity to rapidly absorb amino acids, permitting in transport of plant eating assimilation and improving amino acid obtainability in the digestive system [23]. Despite the potential effects of tannins on food properties, it is important to note that fruits with low tannin content may not pose significant health risks. While tannins have been linked to certain antinutritional effects, such as reduced protein digestibility, the impact of tannins on overall health can vary depending on the quantity consumed and an individual's dietary context [31]. Finally, the content of saponin in this investigation ranged from 0.36 mg/100 g to 2.1 mg/100 g in the fruits of *C. africana* and *E. schimperi*, respectively. Saponins, a class of naturally occurring compounds found in various plant species, play a significant role in shaping the taste and texture of food. When present in substantial quantities, saponins can introduce a characteristic bitter flavor to food items and induce foaming when mixed in an aqueous solution. This article is aimed at delving into the implications of saponins on food properties, exploring their impact on sensory perception and culinary applications [32]. In addition to their impact on red blood cells, saponins are known to exert irritant effects on the gastrointestinal tract. When consumed, 100 mg/100 g saponins can cause gastric irritation, leading to symptoms such as nausea, vomiting, and abdominal pain. This has been attributed to the ability of saponins to disrupt the mucous lining of the digestive system and promote inflammation [33]. Small antinutritional matters could errand nutrient absorption and exploitation in nutrition preparations to relieve protein energy starvation [34].

## 4. Limitation of the Study

Due to limited funds and a well-equipped laboratory, the toxicity, antioxidant activity, fatty acid content, and phytochemical studies of the five wild edible fruit species were not fully completed.

## 5. Conclusion and Recommendation

According to their recommended proximate and mineral contribution to daily nutrient requirements in humans, *E. schimperi*, *C. africana*, *F. vasta*, *M. kummel*, and *S. guineense* are sound in their dietary composition and in combating hunger especially in developing countries. Fruits may not pose significant health hazards due to their low level of antinutrients. The study suggests that wild edible fruits can combat hunger and nutrient deficiencies, despite their limited popularity in Ethiopia. More studies are vital to assess the full nutritional profile and antinutritional quality in these wild edible plants and encourage farmers to cultivate in the agroforestry system.

## Figures and Tables

**Table 1:** Taxonomic information of five selected wild edible plant species in Simada District.

**Plant species**	**Family**
*E. schimperi*	Myrsinaceae
*C. africana*	Boraginaceae
*F. vasta*	Moraceae
*M. kummel*	Sapotaceae
*S. guineense*	Myrtaceae

**Table 2:** Proximate composition of five selected semiwild fruit–bearing edible plants grown in agroforestry of Simada District, South Gondar, Ethiopia.

**Parameters**	***E. schimperi***	***C. africana***	***F. vasta***	***M. kummel***	***S. guineense***
Moisture	11.5 ± 0.03^a^	9.8 ± 0.00^c^	13.5 ± 0.002^a^	12.8 ± 0.00^b^	16.3 ± 0.001^b^
Total ash	2.5 ± 0.01^b^	5.5 ± 0.03^b^	7.8 ± 0.002^a^	3.1 ± 0.00^a^	0.78 ± 0.00^a^
Crude fiber	18 ± 0.03^d^	6.7 ± 0.01^b^	14 ± 0.00^b^	19.5 ± 0.00^b^	3.4 ± 0.02^c^
Crude fat	2.1 ± 0.02^b^	1.1 ± 0.00^d^	3.2 ± 0.01^c^	1.86 ± 0.00^a^	0.5 ± 0.01^a^
Crude protein	6.2 ± 0.03^c^	8.7 ± 0.03^c^	7 ± 0.03^a^	3.2 ± 0.000^a^	2.3 ± 0.01^c^
Carbohydrate	59.7 ± 0.27^d^	64.4 ± 0.03^a^	53.5 ± 0.02^d^	60.22 ± ±0.25^c^	76.72 ± 0.01^d^
Energy	282.5 ± 2.11^d^	302.3 ± 0.12^a^	274.8 ± 0.06^d^	267.7 ± 1.3^c^	320.58 ± 0.03^d^

*Note:* The values are the means of three independent composite sample assessments (based on DW). Variable superscripts in the column signify significant differences at *p* < 0.05.

## Data Availability

The data used to support this study are included in the article.

## References

[1] Kibar B., Temel S. (2016). Evaluation of Mineral Composition of Some Wild Edible Plants Growing in the Eastern Anatolia Region Grasslands of Turkey and Consumed as Vegetable. *Journal of Food Processing and Preservation*.

[2] Jiru N. A., Fekadu Gemede H., Keyata E. O. (2023). Nutritional Composition and Antioxidant Properties of Selected Underutilized Wild Edible Fruits in East Wollega Zone, Western Ethiopia. *International Journal of Fruit Science*.

[3] Abdi F. A., Gemede H. F., Olika Keyata E. (2022). Nutritional Composition, Antinutrient Contents, and Polyphenol Compounds of Selected Underutilized and Some Commonly Consumed Vegetables in East Wollega, West Ethiopia. *Journal of Food Quality*.

[4] Ibrahim O., Menkovska M. (2022). Dietary Fibers Classification, Properties, Analysis and Function: A Review. *Advances in Bioscience and Biotechnology*.

[5] Ioniță-Mîndrican C. B., Ziani K., Mititelu M. (2022). Therapeutic Benefits and Dietary Restrictions of Fiber Intake: A State of the Art Review. *Nutrients*.

[6] Hervik A. K., Svihus B. (2019). The Role of Fiber in Energy Balance. *Journal of Nutrition and Metabolism*.

[7] Seal T., Pillai B., Chaudhuri K. (2017). Evaluation of Nutritional Potential of Five Unexplored Wild Edible Plants Consumed by the Tribal People of Arunachal Pradesh State in India. *Journal of Food and Nutrition Research*.

[8] Wu G. (2016). Dietary Protein Intake and Human Health. *Food & Function*.

[9] Tolessa K., D’heer J., Duchateau L., Boeckx P. (2017). Influence of Growing Altitude, Shade and Harvest Period on Quality and Biochemical Composition of Ethiopian Specialty Coffee. *Journal of the Science of Food and Agriculture*.

[10] Passos B. N., Lima M. C., Sierra A. P. R. (2019). Association of Daily Dietary Intake and Inflammation Induced by Marathon Race. *Mediators of Inflammation*.

[11] Smith J. E. W., Holmes M. E., McAllister M. J. (2015). Nutritional Considerations for Performance in Young Athletes. *Journal of Sports Medicine*.

[12] Yiblet Y., Adamu E. (2023). Nutritional Composition and Phytochemical Evaluation of Some Selected Wild Edible Plants in Tach Gaint District, Northwestern Ethiopia. *Scientific World Journal*.

[13] Yimer A., Forsido S. F., Addis G., Ayelign A. (2023). Nutritional Composition of Some Wild Edible Plants Consumed in Southwest Ethiopia. *Heliyon*.

[14] Bouziani A., Saeid N., Benkirane H. (2018). Dietary Calcium Intake in Sample of School Age Children in City of Rabat, Morocco. *Journal of Nutrition and Metabolism*.

[15] Wrzosek M., Woźniak J., Kozioł-Kaczorek D., Włodarek D. (2019). The Assessment of the Supply of Calcium and Vitamin D in the Diet of Women Regularly Practicing Sport. *Journal of Osteoporosis*.

[16] Owaidah T., Al-Numair N., Al-Suliman A. (2020). Iron Deficiency and Iron Deficiency Anemia Are Common Epidemiological Conditions in Saudi Arabia: Report of the National Epidemiological Survey. *Anemia*.

[17] Yan F., Li K., Xing W., Dong M., Yi M., Zhang H. (2022). Role of Iron-Related Oxidative Stress and Mitochondrial Dysfunction in Cardiovascular Diseases. *Oxidative Medicine and Cellular Longevity*.

[18] Sapkota M., Knoell D. L. (2018). Essential Role of Zinc and Zinc Transporters in Myeloid Cell Function and Host Defense Against Infection. *Journal of Immunology Research*.

[19] Prasad A. S. (2020). Lessons Learned from Experimental Human Model of Zinc Deficiency. *Journal of Immunology Research*.

[20] Silva V. M., Putti F. F., White P. J., Dos Reis A. R. (2021). Phytic Acid Accumulation in Plants: Biosynthesis Pathway Regulation and Role in Human Diet. *Plant Physiology and Biochemistry*.

[21] Abera S., Yohannes W., Chandravanshi B. S. (2023). Effect of Processing Methods on Antinutritional Factors (Oxalate, Phytate, and Tannin) and Their Interaction With Minerals (Calcium, Iron, and Zinc) in Red, White, and Black Kidney Beans. *International Journal of Analytical Chemistry*.

[22] Ferraro P. M., Bargagli M., Trinchieri A., Gambaro G. (2020). Risk of Kidney Stones: Influence of Dietary Factors, Dietary Patterns, and Vegetarian-Vegan Diets. *Nutrients*.

[23] Díaz Carrasco J. M., Cabral C., Redondo L. M. (2017). Impact of Chestnut and Quebracho Tannins on Rumen Microbiota of Bovines. *BioMed Research International*.

[24] Sareen A., Gupta R. C., Bansal G., Singh V. (2020). Comparison of Key Mineral Elements in Wild Edible Fruits of Ziziphus *Mauritiana* and *Z. Nummularia* Using Atomic Absorption Spectrophotometer (AAS) and Flame Photometer. *International Journal of Fruit Science*.

[25] Spiller G. A., Amen R. J., Kritchevsky D. (1975). Dietary Fiber in Human Nutrition. *Critical Reviews in Food Science & Nutrition*.

[26] EFSA Panel on Dietetic Products, Nutrition, and Allergies (NDA) (2010). Scientific Opinion on Dietary Reference Values for Carbohydrates and Dietary Fibre. *EFSA Journal*.

[27] Chasapis C. T., Ntoupa P. S. A., Spiliopoulou C. A., Stefanidou M. E. (2020). Recent Aspects of the Effects of Zinc on Human Health. *Archives of Toxicology*.

[28] Reta H. T., Demissew S., Asfaw Z., Zewdu A. Micronutrient and Phytic Acid Contents of Wild Edible Fruits Collected From Temcha Watershed of Amhara Region (Ethiopia) to Combat Hidden Hunger.

[29] Noonan S., Savage G. P. (1999). Oxalate Content of Foods and Its Effect on Humans. *Asia Pacific Journal of Clinical Nutrition*.

[30] Wafula E. N., Onduso M., Wainaina I. N. (2022). Anti-Nutrient to Mineral Molar Ratios of Raw Common Beans and Their Rapid Prediction Using Near-Infrared Spectroscopy. *Food Chemistry*.

[31] Zhang L., Xu M., Guan Q., Jiang J., Sun K., Manirafasha E. (2023). Determination of Vegetable Tannins From Plants in China. *Biofuels, Bioproducts and Biorefining*.

[32] Lim J. G., Park H. M., Yoon K. S. (2020). Analysis of Saponin Composition and Comparison of the Antioxidant Activity of Various Parts of the Quinoa Plant (*Chenopodium quinoa* Willd.). *Food Science & Nutrition*.

[33] Elgailani I. E. H. (2015). Spectrophotometric and Phytochemical Analysis of Black Tea (*Camellia sinensis* Leaves). *Journal of Applied and Industrial Sciences*.

[34] Olika Keyata E., Tola Y. B., Bultosa G., Fikreyesus Forsido S. (2020). Proximate, Mineral, and Anti-Nutrient Compositions of Underutilized Plants of Ethiopia: Figl (*Raphanus sativus* L.), Girgir (*Eruca sativa* L) and Karkade (*Hibiscus sabdariffa*): Implications for *In-Vitro* Mineral Bioavailability. *Food Research International*.

